# Approaches for the Efficient Removal of Fluoride from Groundwater: A Comprehensive Review

**DOI:** 10.3390/toxics12050306

**Published:** 2024-04-23

**Authors:** Negar Arab, Reza Derakhshani, Mohammad Hossein Sayadi

**Affiliations:** 1 Department of Environmental Engineering, Faculty of Natural Resources and Environment, University of Birjand, Birjand 9717434765, Iran; negar.arab_markade@birjand.ac.ir; 2 Department of Geology, Shahid Bahonar University of Kerman, Kerman 7616913439, Iran; 3 Department of Earth Sciences, Utrecht University, 3584 CB Utrecht, The Netherlands; 4 Faculty of Natural Resources and Environment, Shahid Bahonar University of Kerman, Kerman 7616913439, Iran; mh_sayadi@uk.ac.ir

**Keywords:** human health, VOS Viewer, groundwater remediation, advanced oxidation process

## Abstract

Contamination of groundwater with fluoride represents a significant global issue, with high concentrations posing serious public health threats. While fluoride is a critical element in water, excessive levels can be detrimental to human health and potentially life-threatening. Addressing the challenge of removing fluoride from underground water sources via nanotechnological approaches is a pressing concern in environmental science. To collate relevant information, extensive literature searches were conducted across multiple databases, including Google Scholar, PubMed, Scopus, Web of Science, the American Chemical Society, Elsevier, Springer, and the Royal Society of Chemistry. VOS Viewer software version 1.6.20 was employed for a systematic review. This article delivers an exhaustive evaluation of various groundwater fluoride removal techniques, such as adsorption, membrane filtration, electrocoagulation, photocatalysis, and ion exchange. Among these, the application of nanoparticles emerges as a notable method. The article delves into nano-compounds, optimizing conditions for the fluoride removal process and benchmarking their efficacy against other techniques. Studies demonstrate that advanced nanotechnologies—owing to their rapid reaction times and potent oxidation capabilities—can remove fluoride effectively. The implementation of nanotechnologies in fluoride removal not only enhances water quality but also contributes to the safeguarding of human health.

## 1. Introduction

Groundwater serves as the primary source of drinking water and is utilized by a significant portion of the global population [1]. The contamination of groundwater with fluoride poses a serious issue that impacts millions of people around the world [2,3]. Moreover, the consumption of fluoride-laden groundwater can lead to fluorosis, which is highly dangerous [4]. It is acknowledged that the removal of fluoride from groundwater yields positive outcomes, such as improved dental health, decreased risk of bone diseases, and the prevention of conditions like fluorosis, while also mitigating environmental impacts. However, despite these benefits, the extraction of fluoride can sometimes trigger a series of adverse effects on both human health and the environment [5]. For instance, while diminishing fluoride levels in water can lessen dental diseases, it may conversely lower the resistance of teeth to decay. The consumption of water with low fluoride content can impair the absorption of calcium and phosphorus in the bones, potentially leading to bone-related ailments such as osteoporosis, which is characterized by a decrease in bone density [6]. Furthermore, a reduction in fluoride levels can weaken resistance to bacterial infections. In certain cases, drinking water with insufficient fluoride may lead to the emergence of fluoride-associated diseases, including fluorosis, periodontitis, and even cancer [7].

Removing fluoride groundwater could lead to an increase in other contaminants, causing the water to become highly polluted. The use of methods to remove fluoride from water may cause the production of dangerous waste, which, if not disposed of properly, can harm the environment and animal life [8]. Removing fluoride from water can cause changes in the physical and chemical balance of water, which can be harmful to local organisms and plants. In some cases, the removal of fluoride from water may cause changes in the pH and electro-conductivity of water, which can lead to a decrease in biodiversity and negative effects on aquatic ecosystems. Since fluoride is an important element in the regulation of dental and bone health, its removal from groundwater must be conducted accurately and in compliance with health and environmental standards. To avoid adverse consequences, sustainable and effective fluoride removal methods can be used, and as an alternative, fluoride conservation methods can be used in groundwater. In general, it can be said that the removal of fluoride from underground water should be performed with full care and consideration due to the various consequences that may occur to human health and the environment.

The main issues in removing fluoride from groundwater are its high concentration in water sources and the presence of other mineral compounds [9]. Economical and efficient techniques are needed to remove fluoride at high levels in the presence of other minerals acting as interfering substances [10]. Advanced nanotechnology methods have created the promise of being able to remove fluoride from groundwater under natural conditions with high efficiency, selectivity, rapid action rates, cost-effectiveness, and minimal side effects.

The advantages of utilizing nanotechniques to eliminate fluoride from underground water are articulated; Nano-enhanced methods are effective at removing fluoride from water and reducing its concentration [11]. The removal process is significantly faster with the implementation of these techniques, which reduces the overall time required for purification [12]. The long-term life span of nanotechnology methods often requires the reconstruction of components and equipment, and sometimes their replacement [13]. Simpler equipment that costs less to install and operate is commonly needed for the use of advanced nano-methods [14]. The use of nanometer materials in nano-enhanced methods typically results in fewer side effects on the environment and human health [15]. Considering these advantages, the use of nanotechnology can be considered as a good option to remove fluoride from underground water.

Adsorption or separation are the main mechanisms for fluoride removal by nanotechnology methods [16]. First, in the absorption method, nanomaterials with a high surface area are able to absorb fluoride [17]. This adsorption is usually carried out by electrostatic forces and chemical dissociation [18]. Some common nanomaterials used for fluoride absorption include activated carbon, silica nanoparticles, iron nanoparticles, and zirconium nanoparticles [19]. In the second separation method, active nanoparticles are used as filters to separate fluoride from water [20]. These nanoparticles are usually composed of materials such as iron nanoparticles, alumina nanoparticles, and zirconium nanoparticles [21]. The interaction between fluoride and nanomaterials is usually carried out by electrostatic forces, surface attraction, chemical dissociation, or hydrogen interactions. These interactions cause the adsorption of fluoride by nanomaterials and the separation of the fluoride from the water [22].

Fluoride removal from groundwater requires cost-effective and efficient methods. Various nanotechnologies, including adsorption, membrane filtration, electrocoagulation, photocatalysis, and ion exchange, have been developed over the years [23]. Adsorption, which includes the use of activated carbon, alumina, and other adsorbents, is widely used for fluoride removal [24]. This technique is effective, cost-effective, and does not produce any dangerous by-products [25]. However, adsorption suffers from adsorbent saturation and requires frequent regeneration or replacement [26]. Another method is ion exchange, which is an attractive option for the selective removal of fluoride ions due to its efficiency, high capacity, and ease of operation. Ion-exchange resins are another method that can be used to remove various types of elements from the environment. However, this process is expensive, requires careful maintenance, and produces significant waste. Membrane filtration methods such as reverse osmosis, nanofiltration, and ultrafiltration are very effective in removing fluoride from water. However, these methods are expensive and require high energy input and maintenance [27]. In recent years, nano-methods have been considered as a potential solution to remove fluoride from groundwater [28]. Another promising method for fluoride removal is electrocoagulation. Unlike conventional coagulation methods, electrocoagulation uses a low-voltage direct current to remove fluoride from water. This process is economical, environmentally friendly, and could be applied in a wide range of water systems.

This article conducted a systematic review of different methods for removing fluoride from underground water sources. The PRISMA flow diagram can be found in Supplementary Materials. The researchers utilized databases such as Google Scholar, PubMed, Scopus, Web of Science, American Chemical Society, Elsevier, Springer, and Royal Society of Chemistry for their investigation. VOS Viewer software was applied to analyze and organize the collected data. The effectiveness of various techniques, like adsorption, membrane filtration, electrocoagulation, photocatalysis, and ion exchange, was assessed through systematic studies.

## 2. Adsorption

**Figure 1:**
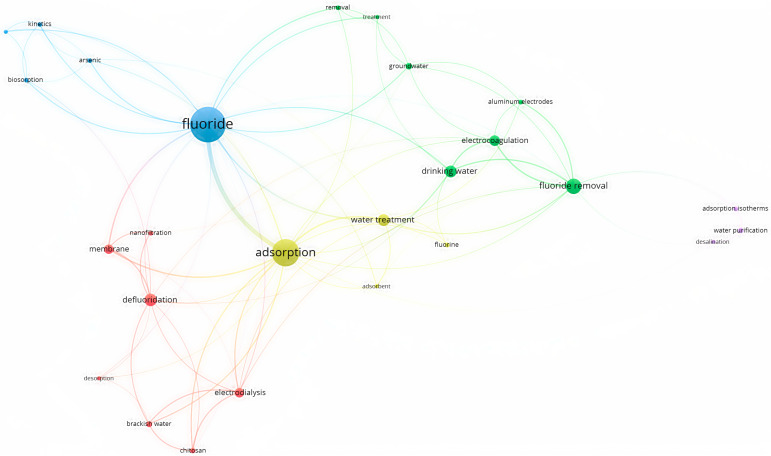
Keyword and overlay visualization of co-occurrence analysis of fluoride adsorption using VOS Viewer.

When the fluoride level in water exceeds the permissible limit (<1.5 mg/L), water pollution becomes a major concern for humans. A co-occurrence analysis of fluoride absorption using VOS Viewer is displayed in Figure 1 through an overlay visualization. As Figure 1 shows, previous studies have made noteworthy contributions to the removal of fluoride from groundwater by surface adsorption techniques. In this method, an adsorbent (for example, natural adsorbents such as zeolite, activated carbon, clay, polymer mixtures, etc.) comes into contact with water containing metals, and the metals are absorbed on the surface of the adsorbent. Then, the adsorbent containing the adsorbed metals is separated from the water, and the metals are used separately for recovery and reuse or proper waste disposal. Various factors, such as the adsorbent type, amount of fluoride, pH, temperature, contact time, and intensity of mixing, affect the absorption efficiency. The adsorption method is widely used as one of the primary methods for removing metals from water and wastewater. It is favored for its simplicity, high operational capability, and productivity. Adsorption can effectively remove metals by utilizing various adsorbents and their affinity for metal ions. This method has been extensively studied and applied in water treatment to address fluoride contamination issues [29].

The sorption technique could be a recommended method for treating fluoridated water because it is inexpensive, has readily available adsorbent, can purify water without polluting it, and is environmentally friendly [30]. Historically, alumina- and aluminum-based sorbents, biosorbents, ion-exchange resins, calcium-based materials, carbon-based sorbents, and polymer-based sorbents have all been studied for the removal of fluoride ions from water/wastewater. Polymer-based composites have recently received much attention due to their simplicity of synthesis and biocompatibility, and they can be used to separate fluoride ions. The sorption method includes the use of a material that has a high affinity for fluoride, such as activated alumina, bone charcoal, etc. The working method is that the underground water passes through a column containing absorbent materials that selectively remove fluoride from the water. The absorbent material can be regenerated by washing it with a strong acid solution, allowing it to be reused for multiple cycles. Especially in areas where fluorosis is a common problem, the absorption method is a useful method to remove this pollution. The nature of fluoride adsorption on some adsorbents, especially clays containing iron, aluminum, and silicon oxides, has been investigated as a background for experimental studies to improve the understanding of fluoride–adsorbent interactions. The adsorbed fluoride ions are likely to be exchanged with structural elements within the adsorbent particles, depending on the chemistry of the solids, or the adsorbed fluoride ions are transported to the internal surfaces of porous materials [31].

Graphene is one of the new materials that has been studied as a fluoride absorber in water. Graphene is a type of carbon material that is formed as a thin sheet of carbon core. This material has unique physical and chemical properties and is known as a strong absorbent for polluting elements such as fluoride in water [32]. It has been modified with methyl and nitrogen atoms to investigate its defluorination properties [33]. Vibrational mode analysis for vacancy-induced coronene has been performed to understand its molecular structure and behavior. On the other hand, graphene is a material that has been studied for its potential use as an adsorbent to remove fluoride from water [34]. This material is synthesized and used in the form of a composite with CeO_2_ nanoparticles supported on activated carbon for efficient removal of fluoride from polluted water [35]. Due to the structure of graphene, its upper surface is very large and can act as a very strong adsorbent surface for fluoride. Also, graphene has good chemical properties that make it selectively absorb fluoride from water and reject other ions and substances in the water. To use graphene as a fluoride adsorbent, it is usually dissolved in water in the form of nanoparticles, and then, using special processes such as filtration, the fluoride adsorbent is separated from the water [36]. Using graphene as a fluoride absorber has advantages and disadvantages. As an advantage, we can point out the extremely high absorption power of graphene. Also, graphene has very high chemical and thermal stability, which makes it able to remove fluoride from water. In addition, graphene is a very light and thin material that can easily be moved, and it also reduces installation and maintenance costs [37]. But as disadvantages, one could mention the high price of graphene production and the need for advanced technology to produce graphene. Sometimes, it can also absorb other elements, such as calcium and magnesium. In addition, if graphene is dissolved in water in the form of nanoparticles, it may cause problems for the environment and human health due to health risks such as toxicity and axiomatization [35,36,37].

**Figure 2:**
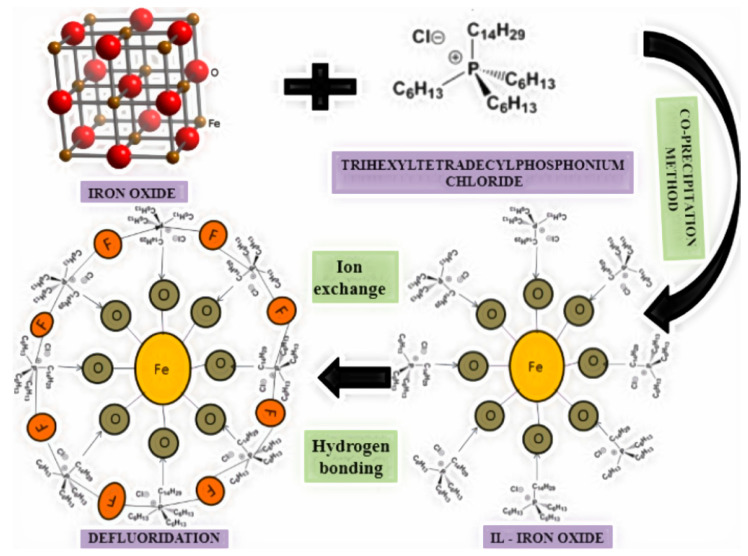
The possible mechanism for removal of fluoride involving ionic liquids–iron oxide [38].

The interaction mechanism between ionic liquids–iron oxide and fluoride ions is depicted in Figure 2. The magnetic properties facilitate the binding of iron oxide and F− ions. The ionic liquids rely on electrostatic attraction to bind with iron oxide. Additionally, trihexyl(tetradecyl)phosphonium chloride contributes to fluoride adsorption through ion-pair attraction, ion exchange, protonated hydroxyl groups, and hydrogen bonding. The OH and carboxyl groups, as well as their electrostatic properties, are crucial in accelerating the absorption of ionic liquids, such as iron oxide [38].

Recently, studies in nanotechnology using nanomaterials as adsorbents in wastewater treatment have received attention [39]. Pure adsorbents have many applications but have limiting properties, such as non-selective adsorption and recycling problems. To expand the scope of application of absorbent materials, the structure is improved and the absorption sites are increased by combining two different absorbent materials with a synergistic effect. In theory, the process of fluoride adsorption onto solid particles comprises three primary stages: initial migration of fluoride from the solution to the external surface of the absorbent, subsequent attachment of fluoride onto the surface of the particle, and eventual ion exchange or diffusion into the particle [40]. To evaluate the possibility of using an adsorbent in practice, one should consider its adsorption capacity in dilute solutions, its pH, the contact time, the stability of the adsorbent, its regeneration, the effect of other anions and cations, and the total costs [41]. The adsorption process provides satisfactory results and is a promising method for fluoride removal from groundwater.

The most popular method for removing fluoride from water is adsorption, and the development of adsorbents is the key to the advancement of adsorption technology [42]. The common fluoride removal adsorbents are categorized into four categories in this review: carbon-based, biopolymers, metal oxides/hydroxides, and other adsorbents. Two approaches to creating new adsorbents are the synthesis of composite materials and the utilization of novel materials. Research on the composite synthesis of many types of conventional adsorbents has increased recently, but not as much as that on the discovery of novel adsorbents for fluoride adsorption. Metal oxides, which were initially used as adsorbents, can be used as active centers in a variety of applications for changing and combining with other types of adsorbents [43].

The current problem of excess fluoride in drinking water around the world makes people look toward low-cost but efficient adsorbents. Recent studies have looked into creating materials that can effectively remove fluoride. So, researchers have developed various adsorbents for this purpose, which are presented in Table 1.

## 3. Membrane Filtration

**Figure 3:**
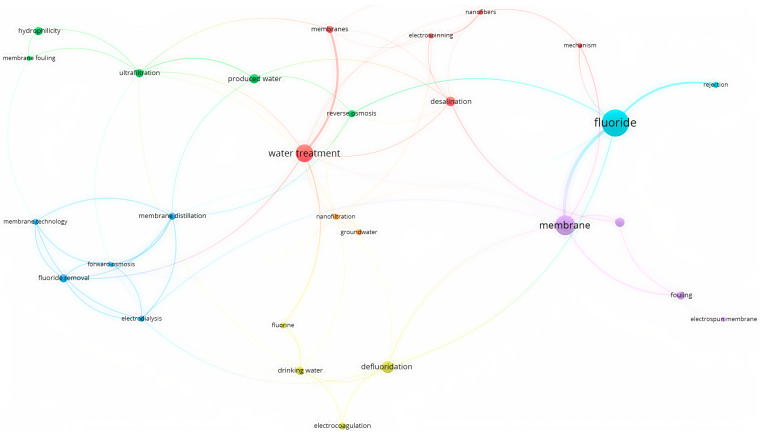
Keyword and overlay visualization of co-occurrence analysis of membrane filtration using VOS Viewer.

Membrane filtration is a method that can be used to remove fluoride from groundwater. This method involves passing water through a semi-permeable membrane that allows water molecules to pass through while trapping fluoride ions. Several membrane filtration methods can be used to remove fluoride, including reverse osmosis, nanofiltration, and ultrafiltration. Each method has different pore sizes and molecular weight cutoffs that determine the size and type of particles that can be removed. A co-occurrence analysis of fluoride membrane filtration using VOS Viewer is displayed in Figure 3 through an overlay visualization.

**Figure 4:**
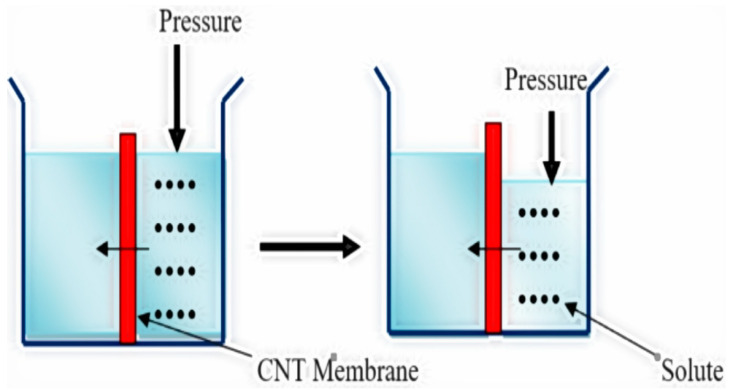
Mechanism of reverse osmosis techniques for water purification [58].

The most common method for removing fluoride ions from groundwater and surface water is the membrane filtration method. This method uses a high-pressure pump to pass water through a semi-permeable membrane that removes fluoride ions and other contaminants. Reverse osmosis membranes have pores of about 0.1 nm and can remove up to 99% of fluoride ions from water. Nanofiltration is another membrane filtration method that can be used to remove fluoride. In this method, a membrane with larger pores than reverse osmosis membranes is used, which enables the removal of larger particles and molecules. Nanofiltration can remove up to 80% of fluoride ions from water [57]. Nanofiltration is broadly divided into two categories: organic solvent nanofiltration and solvent-resistant nanofiltration. Typically, these nanofiltration membranes are composed of cellulose-based and polyamide composite materials, with hollow fiber and spiral wound configurations being common. The operational parameters and longevity of these membranes vary depending on the fabrication techniques. The quality of water obtained from membrane purification depends on factors such as pore size and applied pressure. Reverse osmosis (RO) is a physical separation process where pre-treated water is pressurized and passed through a semi-permeable membrane. In RO, the semi-permeable membrane separates two solutions, with pressure applied to reverse the natural flow of the water. This pressure drives the water from a high concentration compartment to a low concentration compartment. Consequently, impurities and contaminants are excluded and accumulate on one side of the semi-permeable membrane, while pure water is collected on the other side [58]. This process is illustrated in Figure 4. Reverse osmosis is a water purification process based on selectively allowing water molecules to pass through a semi-permeable membrane under pressure while excluding dissolved solutes and contaminants. This selective permeability ensures the production of clean and potable water.

Ultrafiltration is a membrane filtration method with even larger pores that allow the removal of particles such as bacteria and viruses. While ultrafiltration is not usually used solely for fluoride removal, it can be used in combination with other methods to achieve optimal results. Filtering methods may be expensive and require regular maintenance and replacement of membranes, but due to their high efficiency, they are used more than other methods. One of the disadvantages of this type of method is that the membranes can become contaminated over time, and their efficiency decreases, so they need to be cleaned or replaced. As a study, the required pressures are much lower, the energy required is lower, the solute removal is much lower, and the flow is faster [59]. Various studies have been conducted concerning the mechanism of solute retention and the optimization of conditions related to nanofiltration membranes. Solute retention is attributed to steric and charge effects [60]. The subsequent steric effect leads to more retention of fluoride on nanofiltration membranes than competing monovalent anions such as chloride or nitrate [61]. Using hydrogel filters to absorb fluoride from water is one of the methods used in membrane filtration [62]. Hydrogel filters containing zinc oxide nanoparticles are made and used to remove fluoride from groundwater. These filters have removed 97% of the fluoride from water, while only 40% of fluoride is removed in conventional filters. In addition, hydrogel filters containing ZnO nanoparticles remove other chemical pollutants, such as lead, cadmium, and zinc, from water. This method uses membrane filtration with a combination of adsorption and membrane processes. In this method, the groundwater is first filtered through a membrane system to remove large particles and create high pressure. Then, the water is directed to the hydrogel filters containing ZnO nanoparticles. In these filters, ZnO nanoparticles absorb fluoride from the water, and the treated water is re-passed through a membrane filter to remove smaller particles and other chemical contaminants.

## 4. Electrocoagulation

**Figure 5:**
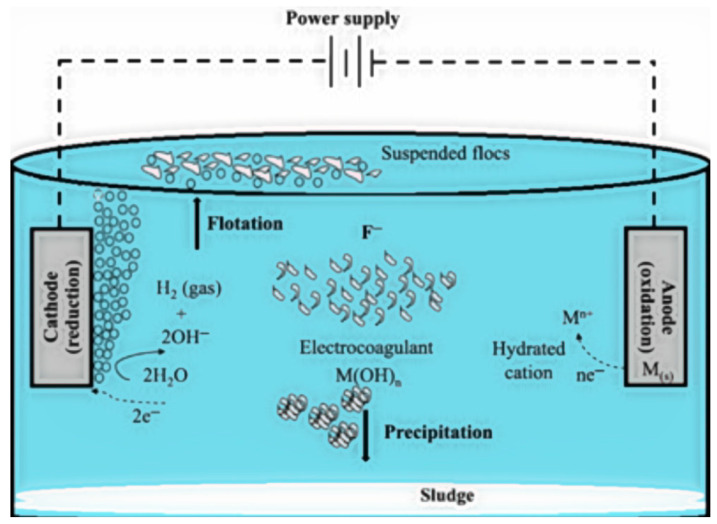
A schematic depiction of a fundamental EC cells operating in batch mode [64].

**Figure 6:**
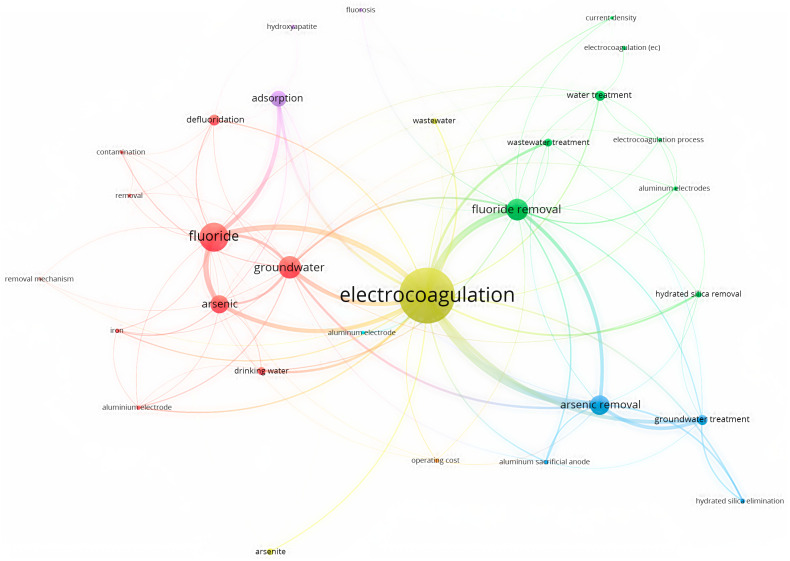
Keyword and overlay visualization of co-occurrence analysis of electrocoagulation using VOS Viewer.

Electrocoagulation (EC) is another method to remove fluoride ions. EC is an electrochemical technique used to remove contaminants from water through physicochemical processes driven by an electrical current. As Figure 5 shows, in a basic EC setup, two metal electrodes, an anode, and a cathode are employed. Oxidation occurs at the anode, producing metallic cations, while reduction takes place at the cathode, forming hydrogen gas bubbles and hydroxide ions. This leads to the neutralization of pollutants and the formation of larger flocs for easier removal. Subsequently, physical separation methods like flotation and filtration are employed to separate the flocs from the water. Inorganic pollutants are attracted to working electrodes due to electrophoresis, aiding in their removal. Despite its advantages, such as simplicity and minimal chemical usage, EC also has drawbacks, like the need for electrode replacement and the formation of passivated films [63]. Figure 5 shows an overlay visualization of a co-occurrence analysis of the electrocoagulation method for the removal of fluoride from underground water. As shown in Figure 6, the electrocoagulation method has made a great contribution to fluoride removal research. The electrocoagulation process involves passing an electric current through two or more metal electrodes, usually made of iron or aluminum, and placing it in water. The electric current causes the corrosion of the metal electrodes and the release of metal ions in the water. These metal ions then react with fluoride ions in the water to form insoluble metal fluoride complexes that can be removed by sedimentation or filtration. The effectiveness of electrocoagulation for fluoride removal depends on various factors, including the type and concentration of metal electrodes used, current density, and water pH. In general, higher current density and lower pH values increase fluoride removal efficiency. Electrocoagulation has several advantages over other fluoride removal methods, including low cost, ease of operation, and the ability to treat a large volume of water. It also produces less sludge than other coagulation methods and reduces production waste.

Fluoride elimination from contaminated groundwater or wastewater can be achieved through the formation of Al(OH)_3−x_F_x_ precipitate via chemical substitution between hydroxide and fluoride ions, provided that there are not enough OH^-^ ions to neutralize the positive charge of the aluminum cation. Studies have shown that employing aluminum electrodes in the EC process is more efficient for fluoride removal compared to iron electrodes. Research on electrode configurations revealed that employing solely aluminum electrodes resulted in the highest efficiency for fluoride removal, reaching 89%. However, this efficiency declined by 8% upon substituting a single working electrode from aluminum to iron. Conversely, utilizing only iron electrodes yielded a notably lower elimination efficiency of 8.1% [64].

However, electrocoagulation also has disadvantages, such as the potential for corrosion of the metal electrodes and the release of other pollutants into the water, the need for regular maintenance and replacement of electrodes, and the potential for high energy consumption if the process is not optimized. Overall, electrocoagulation is a promising method for fluoride removal from groundwater, but it must be optimized and monitored to ensure effective and efficient treatment [65]. Recently, there has been a growing interest in the application of electrocoagulation for the treatment of wastewater containing organic pollutants, heavy metals, and fluoride [66]. Electrocoagulation is a favorable technique in terms of process conditions; usable components in the water could be separated and reused. Electrocoagulation is a technique that uses anode oxidation to produce the coagulant in situ, typically using aluminum or iron. A typical electrocoagulation reactor consists of several electrolytic cells, each containing a cathode and an anode, which can be made of the same or different materials [67]. The electric current (flow of electrons) causes aluminum or iron to be transferred from an anode material to a solution, leading to the formation of Al^3+^ or Fe^2+^. Simultaneously, the evolution of hydrogen gas and the release of hydroxide anions occur in a cathode. The hydroxide anions move towards an anode and form ion pairs with metal cations. These pairs form polymeric hydroxides of the aluminum or iron, i.e., compounds responsible for coagulation [66]. In this process, metal electrodes (such as aluminum) are placed in water, and by applying an electric current, electrocoagulation activity is created, which leads to the formation of flocculants (solids called flocs). These flocculants are deposited on the fluoride in the water, and by using filtration, the fluoride is removed from the water [68]. Aluminum electrodes were used in underground water with high fluoride concentrations, which showed that fluoride removal increases with increasing electric current and pH, and at pH 7 and an electric current of 1.5 ampere/square meter, the fluoride concentration decreased from 3.2 mg/L to below 1.5 mg/L [69]. Various metal electrodes, such as aluminum, iron, and steel, have been used, and the results have shown that the electrocoagulation process can be used as one of the effective and reliable methods to remove fluoride from groundwater [70].

## 5. Photocatalysis

**Figure 7:**
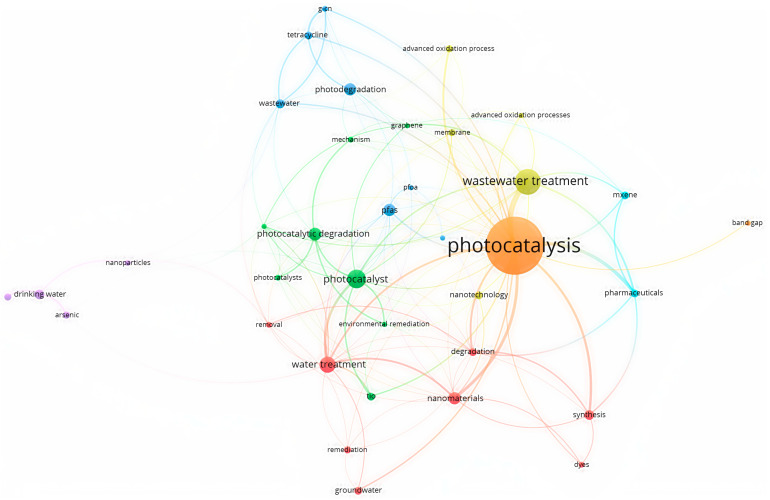
Keyword and overlay visualization of co-occurrence analysis of photocatalysis using VOS Viewer.

Figure 7 shows an overlay visualization of a co-occurrence analysis of photocatalysis using VOS Viewer software, which shows the contribution of different studied parameters related to the study of fluoride removal from water through photocatalysis techniques. This method involves the use of a photocatalyst to remove fluoride ions from water. The photocatalytic process involves the absorption of light by the photocatalyst, which creates electron-hole pairs that react with water molecules to produce OH [71].

The effectiveness of photocatalysis for fluoride removal depends on various factors, such as the type and concentration of photocatalyst used, light intensity and wavelength, and water pH. In general, higher light intensity and lower pH values increase fluoride removal efficiency [72]. Photocatalysis has several advantages over other fluoride removal methods, including its ability to decompose other pollutants in addition to fluoride, its low cost, and its ability to treat a large volume of water. It also does not require the addition of any chemicals, and this method is environmentally friendly. However, photocatalysis also has disadvantages, such as the need for a continuous supply of light, which is difficult to maintain in some settings. In addition, the catalyst can become inactive over time, and its efficiency decreases, so it needs to be replaced. Overall, photocatalysis is a promising method for fluoride removal from groundwater but must be carefully optimized and monitored to ensure effective and efficient treatment [73].

**Figure 8:**
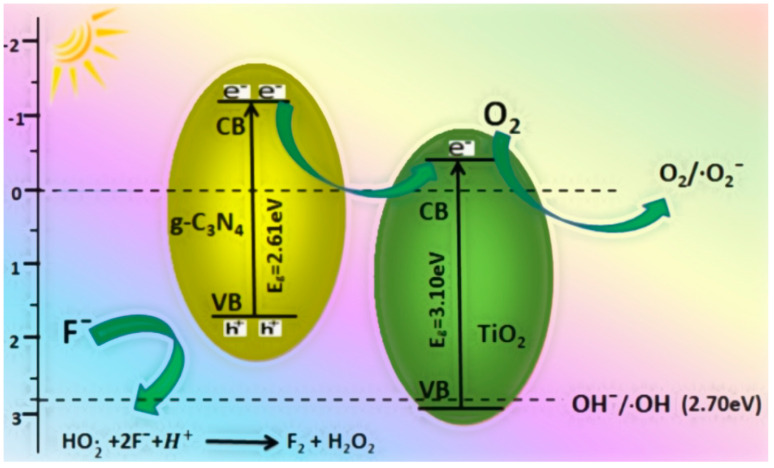
Schematic diagram of the mechanism of photocatalytic degradation of fluoride using g-C_3_N_4_/TiO_2_ photocatalyst under light irradiation [72].

The photocatalytic mechanism in the g-C_3_N_4_/TiO_2_ system involves several key steps to enhance catalytic efficiency. Initially, due to rapid electron-hole pair recombination, g-C_3_N_4_ alone exhibits low catalytic activity. However, when TiO_2_ composite is loaded onto the g-C_3_N_4_, additional active sites for hydrogen evolution are provided, accelerating electron transfer and enhancing the overall catalytic activity. Under visible light irradiation, heterogeneous g-C_3_N_4_/TiO_2_ absorbs photon energy, generating photogenerated electron-hole pairs more efficiently due to its large specific surface area. This prevents the recombination of electron-hole pairs and facilitates their involvement in catalytic reactions. The conduction band and valence band potentials of g-C_3_N_4_ and TiO_2_ play crucial roles in electron transfer. Electrons readily transfer from the conduction band of g-C_3_N_4_ to that of TiO_2_. These electrons then react with adsorbed oxygen, producing highly reactive superoxide radicals (O^2−•^), which combine with water to form hydroxyl radicals (OH^•^). Although the valence band of g-C_3_N_4_ lacks sufficient H+ to react with water molecules, the valence band of TiO_2_ readily combines with water to generate active hydroxyl radicals. Ultimately, these active oxygen groups can adsorb fluoride ions and oxidize them to form F_2_, contributing to fluoride elimination (Figure 8). Overall, the g-C_3_N_4_/TiO_2_ system demonstrates improved photocatalytic efficiency through enhanced electron transfer, increased active sites, and efficient generation of reactive oxygen species under visible light irradiation, facilitating fluoride removal from contaminated water sources [72].

In this process, the interaction of the light and the catalyst is used to produce oxygen free radicals that act as strong oxidizers and remove fluoride from the water by destroying it. A TiO_2_ catalyst was used to remove fluoride from groundwater. The results showed that the fluoride removal increases with increasing time and light intensity, and in optimal conditions, the fluoride concentration decreased from 2.5 mg/L to below 1.5 mg/L. Various catalysts, such as Fe, ZnO, and CdS, have been used, and the results have shown that the photocatalysis process can be used as an effective and reliable method to remove fluoride from groundwater. ZnO and Fe_2_O_3_ catalysts are not sufficient to remove fluoride, but the efficiency of fluoride removal was enhanced when they were combined into a nanocomposite [73,74].

## 6. Ion Exchange

**Figure 9:**
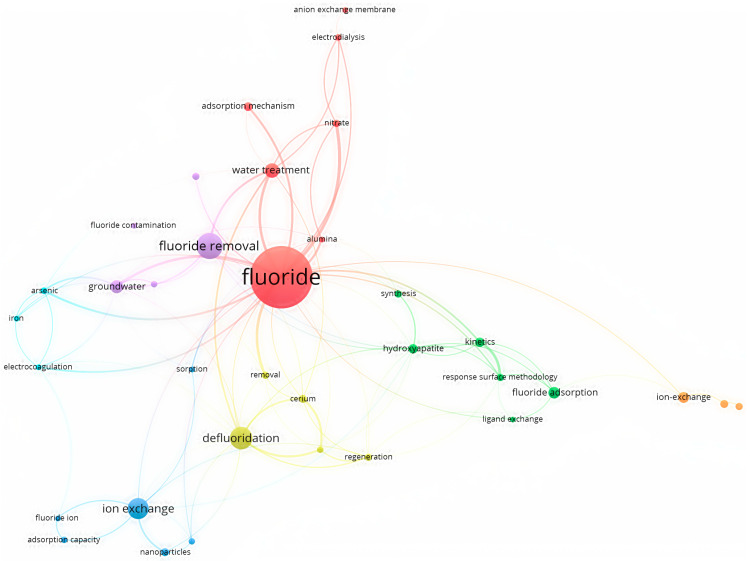
Keyword and overlay visualization of co-occurrence analysis of ion exchange using VOS Viewer.

Ion exchange involves using a resin that exchanges the fluoride ions in the water with other ions, usually chloride or hydroxide ions, which are less harmful [75]. The ion-exchange process involves passing water through a column packed with resin with a high affinity for fluoride ions. As the water passes through the column, fluoride ions are absorbed into the resin and exchanged with other ions. The resin becomes saturated with fluoride ions over time and must be regenerated or replaced to continue removing fluoride from the water [76]. Figure 9 shows an overlay visualization of a co-occurrence analysis of ion exchange using VOS Viewer. As Figure 5 shows, the contribution of the ion-exchange method in studies related to fluoride removal from water was lower than other methods. The effectiveness of ion exchange for fluoride removal depends on various factors, such as the type and concentration of resin used, water flow rate, and water pH [77].

**Figure 10:**
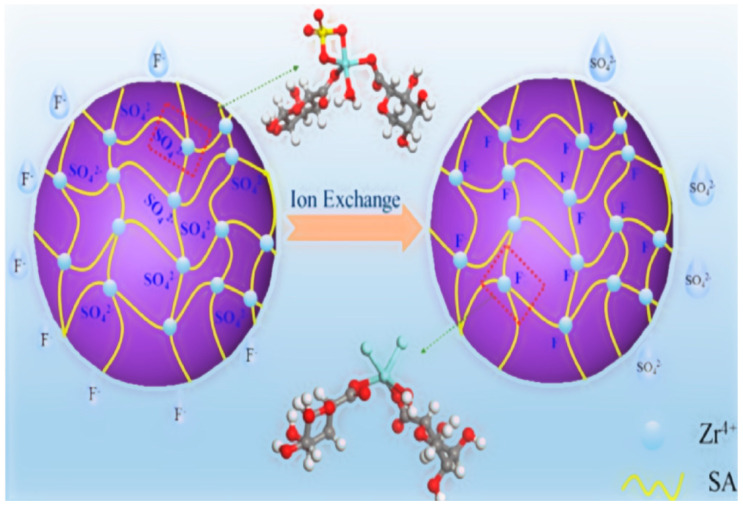
The mechanism of fluoride removal from aquatic environments using ion-exchange techniques [79].

Ion exchange has several advantages over other fluoride removal methods, including its ability to remove fluoride to very low levels, its low operating costs, and its ability to treat large volumes of water [78]. However, ion exchange also has disadvantages, such as the need for regular regeneration or resin replacement, which can be expensive and time-consuming. In addition, the ion-exchange process can be affected by other ions in water that can compete with fluoride ions for exchange sites on the resin [79]. Ion-exchange columns have been used to remove fluoride from groundwater. In this example, Diacon ion-exchange resin is used to absorb fluoride ions in water. Water with fluoride is injected from the top of the column, and the fluoride ions are replaced by other ions in the resin to separate the fluoride from the water. Then, the outlet water from the ion-exchange column is obtained with a lower concentration of fluoride [78,79,80]. Yu and colleagues attempted to synthesize sulfate-type zirconium alginate hydrogel beads with a 3D network structure by introducing sulfate groups to zirconium sites on the surface. As Figure 10 shows, this modification resulted in a porous structure with abundant macropores and mesopores. The sulfate-type zirconium alginate hydrogel beads with a 3D network structure exhibited improved defluorination capability across a wide pH range of 3–9 compared to the synthesized material. Remarkably, these beads demonstrated a maximum adsorption capacity of 101.3 mg/g, surpassing that of other millimeter-scale and some nano-scale adsorbents [79].

Table 2 lists the materials used, treatment capacity, efficiency, and treatment method. It shows that the different removal methods have different efficiencies depending on their conditions, but in general, all these methods show remarkable efficiency for removing fluoride from underground water.

## 7. Conclusions

Recent research has indicated that employing nanotechnology for the removal of fluoride from subterranean water constitutes a novel and effective approach to water purification. In these methods, nanoparticles such as iron, titanium dioxide, activated carbon, and graphene serve as absorbents for fluoride. Owing to their expansive surface area, these nanoparticles are capable of entirely adsorbing fluoride from the water. Given that fluoride may occur naturally in underground water and pose risks to human health, the adoption of nanotechnology for its removal is of significant importance. These methods are not limited to fluoride absorption; they can also eliminate a variety of other chemical pollutants. Furthermore, the small size of nanoparticles and their propensity for uniform distribution in nanotechnologies contribute to enhanced efficiency and performance improvement. Nonetheless, the application of nanotechnologies necessitates further comprehensive research and extensive physical and chemical testing to unequivocally confirm their efficacy. This article also examined the positive and negative impacts of fluoride’s presence or absence in subterranean water, revealing that fluctuations in fluoride levels can adversely affect both the earth and human health. Given the potent capabilities of nanotechnologies in fluoride removal, it is conceivable that these methods will play a crucial role in the future of underground water purification and quality enhancement.

## 8. Future Scope and Research

The future looks bright for using nanotechnology to remove fluoride from groundwater, but more research is needed. Advanced nanomaterials could be further explored for the targeted removal of fluoride. This involves investigating new methods of synthesis, surface modifications, and composite materials to enhance adsorption capacity, selectivity, and reusability. Further investigation is required to enhance the operational parameters of nanotechnology-driven water treatment systems, including flow rates, contact times, and pH conditions. Understanding the kinetics and mechanisms of fluoride adsorption onto nanoparticles can lead to more efficient and cost-effective purification processes. Investigating the long-term stability and performance of nanomaterials under various environmental conditions is crucial for assessing their practical applicability. Research on nanoparticle-based adsorbents will provide insights for sustainability. To ensure their sustainability, it is crucial to assess the potential environmental impact of nanotechnology-based water purification methods. Research in the near future focus on examining the fate, transport, and ecological consequences of nanomaterials in aquatic environments, as well as their potential interactions with other pollutants and organisms. To move from laboratory-scale experiments to real-world applications, it is necessary to collaborate across disciplines and innovate technologically to scale up nanotechnology-based water treatment processes. Research efforts could focus on developing scalable manufacturing methods, modular treatment units, and integration strategies with existing water infrastructure. To ensure widespread adoption, it is crucial to address the cost-effectiveness and accessibility of nanotechnology-based water purification technologies, especially in resource-limited settings. The use of low-cost precursors, the recycling of nanomaterials, and decentralized treatment approaches can all be explored as cost-reduction strategies in future research. To ensure safety, efficacy, and compliance with regulatory requirements, it is necessary to establish regulatory frameworks and quality standards for nanotechnology-based water purification technologies. Addressing regulatory gaps, standardizing testing protocols, and facilitating technology transfer and commercialization could be the focus of future research. Nanotechnology research for fluoride removal from groundwater should focus on innovation, sustainability, and scaling in order to effectively address global water challenges. The field will only progress if scientists, engineers, policymakers, and stakeholders collaborate across disciplines to translate research findings into practical solutions for water purification and quality enhancement.

## Figures and Tables

**Table 1:** Recent adsorbents and their respective fluoride removal capacities.

Adsorbent	Fluoride Removal Capacity (mg/g)	Reference
Mg-Fe-La	112.17	[44]
Polygonum orientale Linn. (Al_2_(SO_4_)_3_ modification)	0.77	[45]
Fe-Al-La	8.17	[46]
CeO_2_/Al_2_O_3_	50	[47]
Fe_3_O_4_/CS/Al(OH)_3_	76.63	[48]
MIL-24(Al)-NH_2_	1070.6	[49]
Al-Cu oxide nanoparticles supported on steel slag	89.5	[50]
LaP-POT(lanthanum phosphate and poly-otoluidine)	10.94	[51]
Iron ore	1.45	[52]
Dolomite	0.011	[53]
Acid-modified pyrolusite (PA-2)	0.58	[54]
Li/Al-LDH	35.4	[55]
Fe-La-Ce	303.03	[56]

**Table 2:** Comparative evaluation of removal efficiency of various processes to remove fluoride from underground water.

Purification Method	Purification Efficiency	Removal Capacity	Materials Used	Process Description
Adsorption	Efficiency varies with materials and parameters. Modified diatomite with aluminum hydroxide can remove up to 98% of fluoride [80].	Maximizing the adsorption capacity of substances in the solution enhances purification potential [81].	Activated carbon, zeolites, ion-exchange resins, membranes [82].	Adsorbent materials attract and sequester organic substances, chemicals, and ions from the solution [83].
Electrocoagulation	Efficiency depends on the current type, electrodes, and other factors. Up to 99% fluoride removal with alternating current and polarity inverter [84].	Successful reduction in fluoride to less than 1.5 mg/L, meeting WHO and TBS standards, with operational expenses between 0.20 and 0.26 EUR per cubic meter [85].	Metal electrodes, semiconductor electrodes, polymer electrodes [86].	Application of electric current removes ions and chemicals from the solution [87].
Membrane Filtration	Up to 99% fluoride removal with reverse osmosis, 95% with electrodialysis, and 90% with nanofiltration [88].	Suitable membranes can treat several hundred to thousand gallons of water daily [89].	Nanofiltration membranes, stomatal membranes, reverse osmosis membranes [88].	Pressure-driven process through membranes isolates and purifies dissolved substances [90].
Photocatalysis	Modified zeolite and Al3+ amended mine waste, removing up to 98% and 80% of fluoride, respectively. Zirconium-based substances and 2D MIL-53(Al) are also effective [84,91].	Efficacy influenced by materials used, with compliance to EPA’s limit of less than 4 mg/L of fluoride in treated water [7].	Photocatalytic nanoparticles, like titanium dioxide, zinc oxide, and zinc sulfide [92].	Activation by visible/ultraviolet light triggers photocatalysts to refine substances [93].
Ion Exchange	Strong-base anion-exchange resins with quaternary ammonium groups remove 90–95% of fluoride but may alter pH and chloride levels [94].	Activated alumina has superior selectivity in fluoride removal, with a capacity of up to 2 kg/cubic foot and an optimal service flow rate of 1 gallon/minute per cubic foot [95].	Ion-exchange resins [96].	Ion-exchange resin assimilates ions from the solution, producing a clear, refined liquid [94].

## Data Availability

The data that support the findings of this study are available upon request.

## Supplementary Materials

The following supporting information can be downloaded at: https://www.mdpi.com/article/10.3390/toxics12050306/s1, Figure S1: The PRISMA flow diagram.
