# Supplementary figures and images for: Approaches for the Efficient Removal of Fluoride from Groundwater: A Comprehensive Review
Source: Toxics. 2024 Apr 23;12(5):306. doi: 10.3390/toxics12050306 (PMC11126082; doi:10.3390/toxics12050306)

## Identification of studies via databases and registers

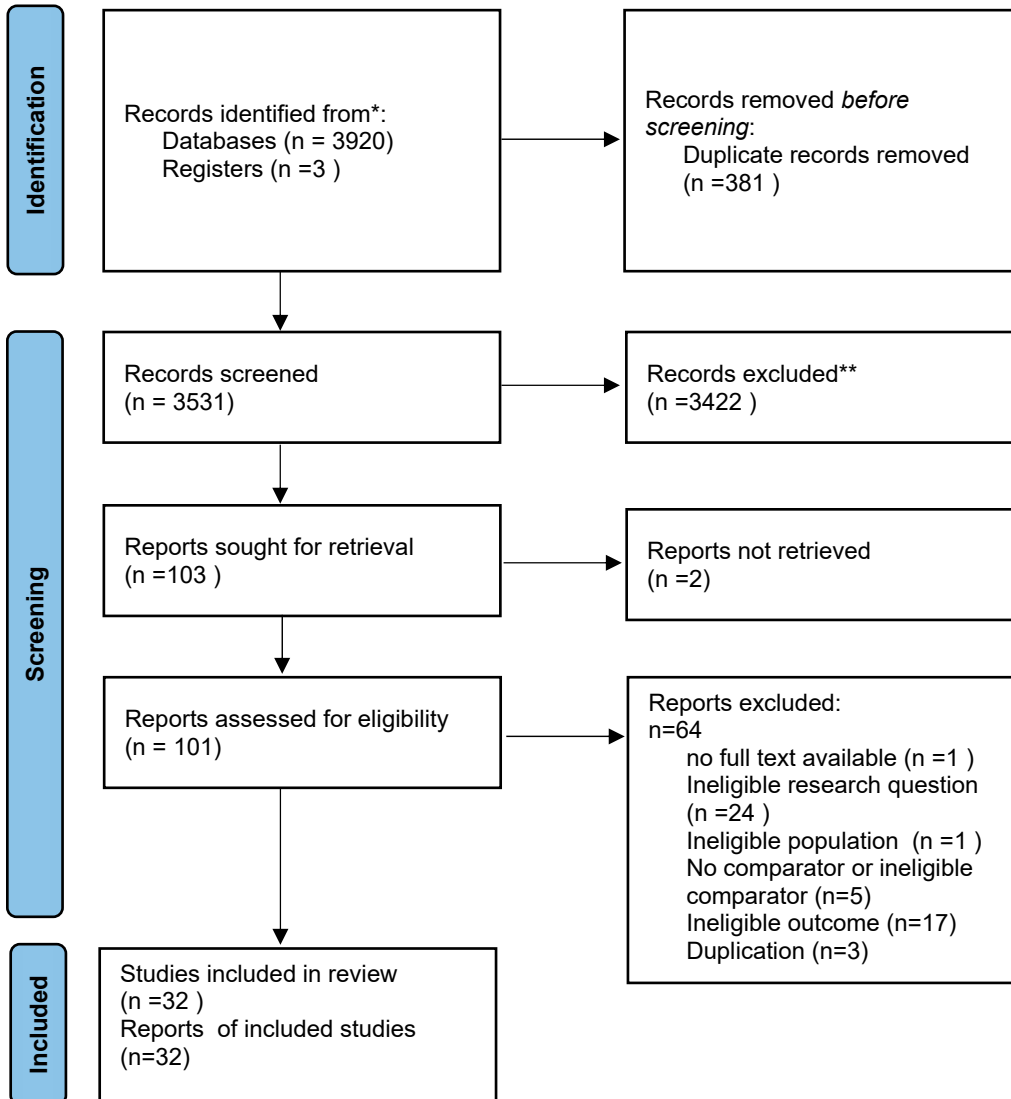

Supplement: Supplementary file 1 [file toxics-12-00306-s001.zip › toxics-2933813-supplementary.pdf]
